# Supplementary material for: Low Prevalence of HER2-Positive Breast Carcinomas among Screening Detected Breast Cancers
Source: Cancers (Basel). 2020 Jun 15;12(6):1578. doi: 10.3390/cancers12061578 (PMC7352518; doi:10.3390/cancers12061578)
Supplement: Supplementary file 1 [file cancers-12-01578-s001.pdf]

Supplementary Material: Low Prevalence of HER2-Positive Breast Carcinomas among Screening Detected Breast Cancers

M. Ángeles López-García, Irene Carretero-Barrio, Belén Pérez-Míes, Miguel Chiva, Carolina Castilla, Begoña Vieites and José Palacios

Table S1. Disease-free survival in the age range 50–69-year-old.

	No of Patients	Event, *n* (%)	Mean Time to Relapse (months), (CI 95%)	*p* [a]
Size (pT)				
T1	452	36 (7.9)	128.5 (126.1–130.9)	<0,0001
T2	268	50 (18.6)	116.9 (112.1–121.7)	
T3-T4	46	23 (50)	79.9 (63.2–96.5)	
Node involvement (pN)				
N0	472	33 (6.9)	128.9 (126.5–131.6)	<0.0001
N1	173	26 (15)	120.1 (114.8–125.4)	
N2.3	124	52 (41.9)	93.9 (84.7–103.1)	
Stage				
I-II	636	53 (8.3)	127.8 (125.7–130)	<0.0001
III-IV	139	60 (43.1)	91.6 (82.7–100.4)	
Grade				
1-2	564	67 (11.8)	125.6 (123–128.2)	<0.0001
3	212	47 (22.1)	108.9 (102.9–114.8)	
LVI				
No	576	62 (10.7)	125.6 (123.1–128.1)	<0.0001
Yes	199	52 (26.1)	109.2 (102.6–115.8)	
SDBC	306	28 (9.1)	126.7 (123.7–129.8)	<0.0001
NSDBC	470	86 (18.2)	118 (114.4–121.7)	
ER				
Positive	626	80 (12.7)	124.8 (122.3–127.3)	0.001
Negative	150	34 (22.6)	109.5 (108.7–117.2)	
PR				
Positive	527	66 (12.5)	125.1 (122.3–127.8)	0.007
Negative	249	48 (19.2)	114.6 (109.2–120)	
HER2				
Positive	104	24 (23.07)	111.06 (102.3–119.8)	0.007
Negative	672	90 (13.4)	123.6 (121.04–126.2)	
Luminal A	361	31 (8.5)	128.4 (125.7– 131)	<0.0001
Luminal B	220	37 (16.8)	120.9 (116.1– 125.7)	
Luminal HER2	45	12 (26.6)	105.5 (92.3–118.1)	
HER2	59	12 (20.3)	112.6 (101–124.3)	
TN NOS	46	10 (21.6)	108.5(98.1–122)	
Basal	45	11(24.4)	100.9 (86.2–115.6)	

[a] *p* values from Mantel-Cox Log-rank test.; CI: confidence interval; LVI: lymphovascular invasion; Luminal A: ER/PR-positive. Ki67≤15%. HER2-negative; Luminal B: ER/PR-positive. Ki67 > 15%. HER2-negative; Luminal HER2: ER/PR-positive. HER2-positive; HER2: ER/PR-negative. HER2 positive; TN NOS: ER/PR-negative. HER2-negative. CK5/6/CK17/CK14/EGFR-negative; Basal: ER/PR-negative. HER2-negative. CK5/6/CK17/CK14 and/or EGFR-positive.

Table S2. Concordance analysis between HER2 status in both hospitals.

		HUVR					
		**No of Cases**	**0. *n* (%)**	**1. *n* (%)**	**2. *n* (%)**	**3. *n* (%)**	***p*** [a]
HURyC	0	27	26 (44.8)	0	1 (1.7)	0	<0.0001
	1	6	0	5 (8.3)	1 (1.7)	0	
	2	4	0	0	1 (1.7)	3 (5.2)	
	3	21	0	0	0	21 (35)	

[a] Measurement of agreement Kappa; HUVR: Hospital Universitario Virgen del Rocío. HURyC: Hospital Universitario Ramón y Cajal; the percentages shown are with respect to the total number of cases.

Table S3. Ki67 differences between the subrogate phenotypes in the series (NSDBC and SDBC) (PDP).

	**No of Patients**	**Ki67 ≤ 15%. *n* (%)**	**Ki67 16–30%. *n* (%)**	**Ki67 > 30%. *n* (%)**	***p*** [a]
Luminal A	361	358 (98.9)	3 (1.1)	0	<0.0001
Luminal B	220	0	183 (68.3)	37 (25.3)	
HER2-positive	104	3 (0.8)	54 (20.1)	47 (32.2)	
TN NOS	46	1 (0.3)	17 (6.3)	28 (19.2)	
Basal	45	0	11 (4.1)	34 (23.3)	

[a] *p* value from Chi-square. Luminal A: ER/PR-positive. Ki67 ≤ 15%. HER2-negative; Luminal B: ER/PR-positive. Ki67 > 15%. HER2-negative; HER2-positive: ER/PR positive or negative. HER2-positive; TN NOS: ER/PR-negative. HER2-negative. CK5/6/CK17/CK14/EGFR-negative; Basal: ER/PR-negative. HER2-negative. CK5/6/CK17/CK14 and/or EGFR-positive

Table S4. Cox model for histopathologic features for disease-free survival and cancer-specific free survival within the HER2-positive breast cancer tumors.

	**Disease-Free Survival**			**Cancer-Specific Free Survival**		
	**Hazard Ratio**	**CI 95%**	***p*** [a]	**Hazard Ratio**	**CI 95%**	***p*** [a]
Size (pT)						
T1-2	1			1		
T3-4	4.06	1.47–11.1	0.007	8.18	1.0–34.5	0.004
Stage						
I-II	1			1		
III-VI	7.28	2.01–8.95	0.007	10.5	7.65–38.7	0.001
SDBC (PDP)	1			1		
NSDBC	1.77	0.50–6.22	0.37	1.23	0.13–8.15	0.85

[a] Cox's proportional hazards modelling; CI: confidence interval.

Table S5. Antibodies.

Serie	Marker	Antibody	Company	Concentration	Positive Scoring Criteria
PDP	ER	Anti- ER (SP1) Rabbit Monoclonal	Roche	Ready to use	>1%
	PR	Anti-PR (1E2) Rabbit Monoclonal	Roche	Ready to use	>1%
	Ki67	Anti-ki67 (30-9) Rabbit Monoclonal	Roche	Ready to use	>15%
	HER2	Pathway anti-HER-2 (4B5) Rabbit Monoclonal	Roche	Ready to use	Wolff et al. [26]
	CK5/6	Anti-Cytokeratin 5/6 (D5/16B4) Mouse Monoclonal	Roche	Ready to use	>20%
	CK14	Anti-Cytokeratin 14 (LL002) Rabbit Monoclonal	Roche	Ready to use	>20%
	CK17	Anti-Cytokeratin 17 (SP95) Rabbit Monoclonal	Roche	Ready to use	>20%
	EGFR	Anti-Epidermal Growth Factor Receptor (3C6) Mouse	Roche	Ready to use	>20%
DEPRECAM	ER	Clone EP1 Rabbit Monoclonal	Agilent-Dako	Ready to use	>1%
	PR	Clone PgR 1294 Mouse Monoclonal	Agilent-Dako	1:50	>1%
	HER2	HercepTest Rabbit Monoclonal	Agilent-Dako	Ready to use	Wolff et al. [26]

PDP: Programa de Detección Precoz del Cáncer de Mama (Andalusia). DEPRECAM: Detección Precoz del Cáncer de Mama (Madrid).

Table S6. HER2 fluorescence in situ hybridization (FISH) used in PDP and DEPRECAM.

Product	Company	Scoring Criteria
PathVysion HER-2 DNA Probe Kit (PathVysion Kit)	Abbot Molecular	Wolff et al. [26]

PDP: Programa de Detección Precoz del Cáncer de Mama (Andalusia). DEPRECAM: Detección Precoz del Cáncer de Mama (Madrid).

© 2020 by the authors. Submitted for possible open access publication under the terms and conditions of the Creative Commons Attribution (CC BY) license (http://creativecommons.org/licenses/by/4.0/).
